# Supplementary figures and images for: Myosin 1C isoform A is a novel candidate diagnostic marker for prostate cancer
Source: PLoS One. 2021 May 21;16(5):e0251961. doi: 10.1371/journal.pone.0251961 (PMC8139512; doi:10.1371/journal.pone.0251961)

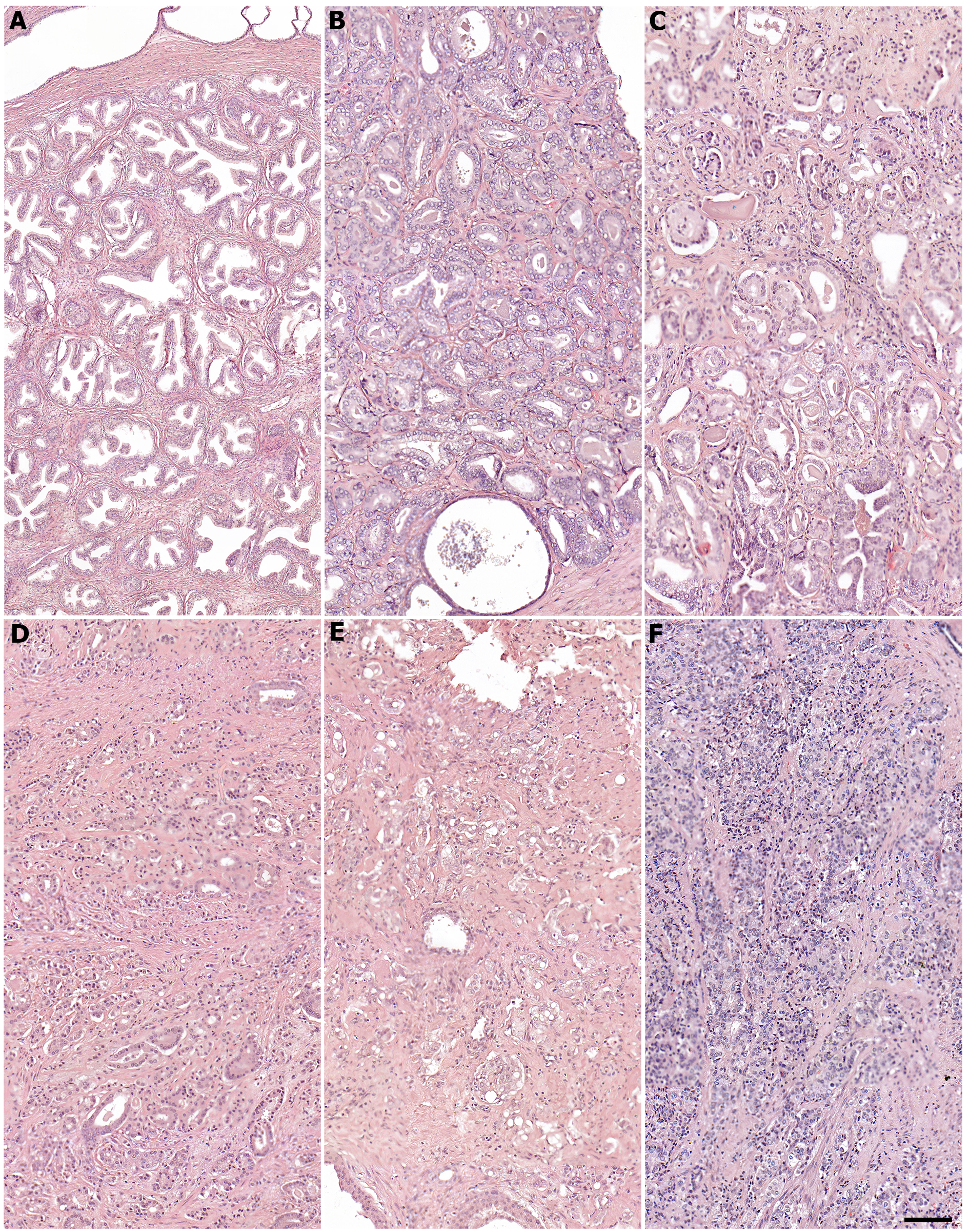

Supplement: S1 Fig — (TIF) [file pone.0251961.s001.tif]

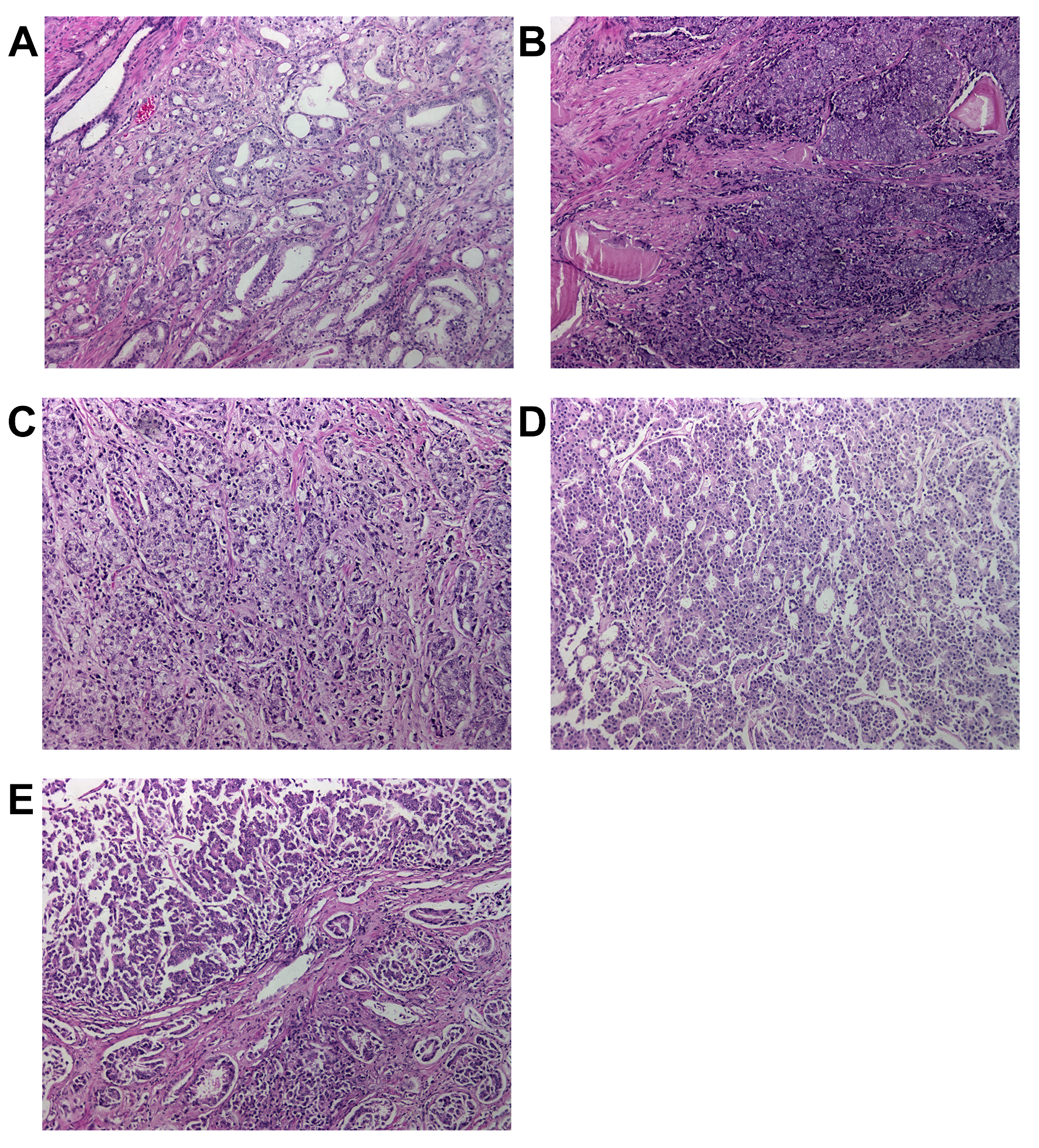

Supplement: S2 Fig — (TIF) [file pone.0251961.s002.tif]

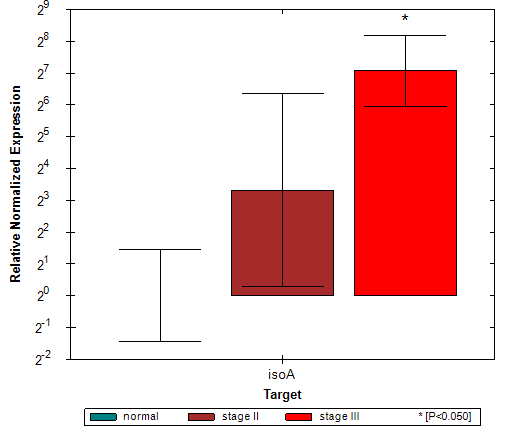

Supplement: S3 Fig — Normalized to total myosin IC. Error bars: standard error of the mean. (TIF) [file pone.0251961.s003.tif]

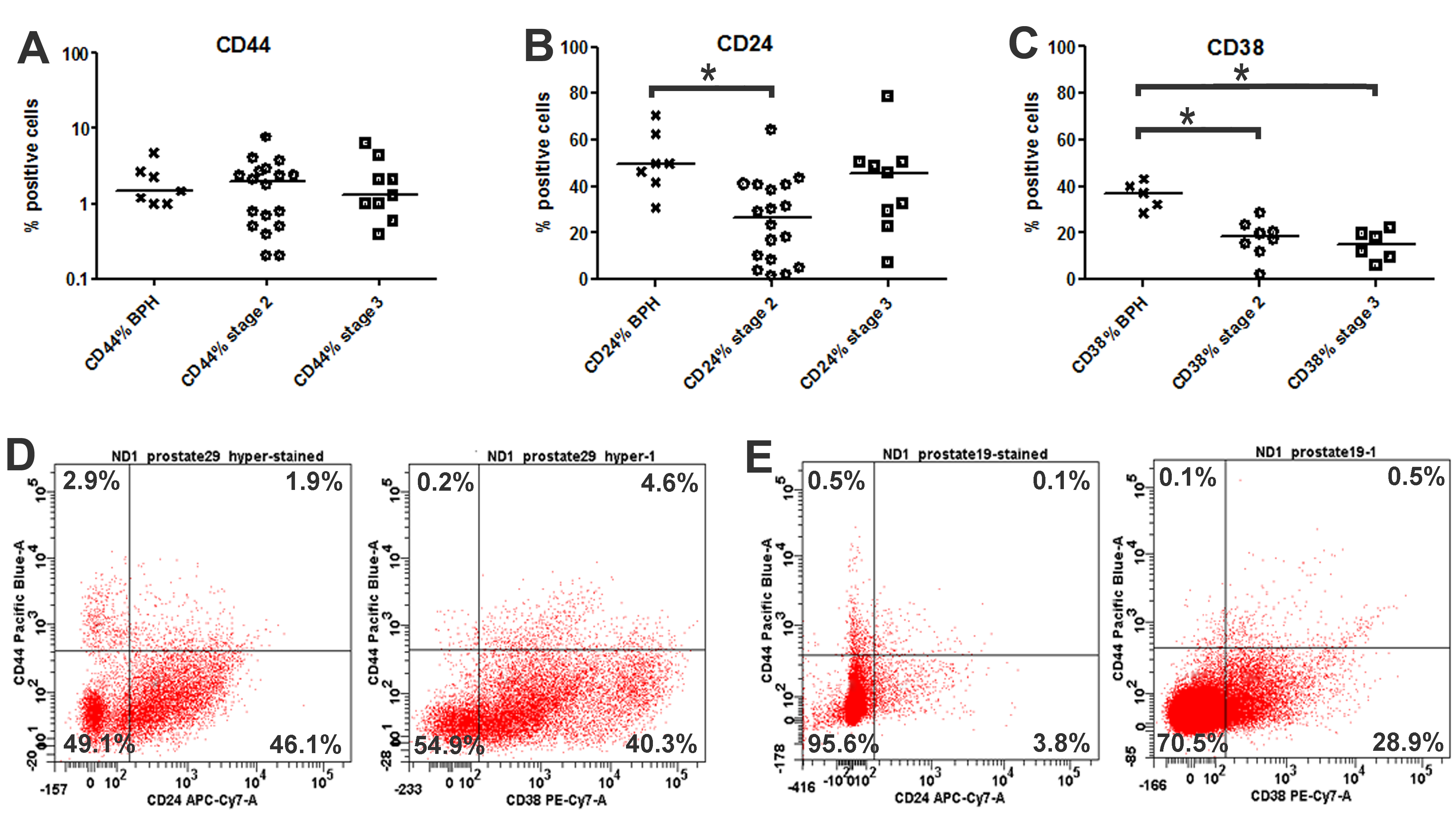

Supplement: S4 Fig — (TIF) [file pone.0251961.s004.tif]

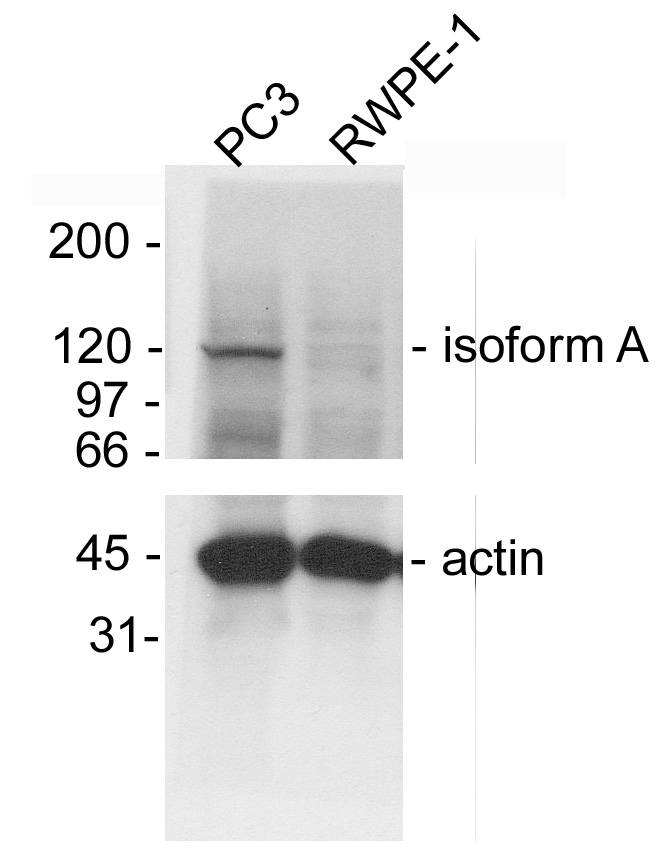

Supplement: S1 Raw image — (JPG) [file pone.0251961.s009.jpg]
