# Supplementary material for: Myosin 1C isoform A is a novel candidate diagnostic marker for prostate cancer
Source: PLoS One. 2021 May 21;16(5):e0251961. doi: 10.1371/journal.pone.0251961 (PMC8139512; doi:10.1371/journal.pone.0251961)
Supplement: S1 Table — (DOCX) [file pone.0251961.s005.docx]

**S1 Table. Clinical data for 41 prostate cancer specimens and 11 specimens with benign prostate hyperplasia (BPH), n/d – not determined**

|  | description | PSA, ng/ml | age | Gleason score |
| --- | --- | --- | --- | --- |
| Specimen 1 | BPH | 1.8 | 67 | 0 |
| Specimen 2 | pT2C | 7.4 | 67 | 3+3=6 |
| Specimen 3 | T3a | 12.7 | 71 | 3+3=6 |
| Specimen 4 | pT2c | 10.5 | 64 | 3+3=6 |
| Specimen 5 | pT3a | 15 | 72 | 4+3=7 |
| Specimen 6 | pT2c | 0.8 | 71 | 3+3=6 |
| Specimen 7 | pT2c | 5.3 | 56 | 4+3=7 |
| Specimen 8 | рТ2b | 6.5 | 56 | 3+4=7 |
| Specimen 9 | рT3b | 16 | 46 | 4+4=8 |
| Specimen 10 | pT2c | 2.7 | 60 | 3+4=7 |
| Specimen 11 | pT2c | 5.4 | 56 | 3+3=6 |
| Specimen 12 | BPH | n/d | 48 | 0 |
| Specimen 13 | pT2b | 5.4 | 48 | 3+3=6 |
| Specimen 14 | BPH | n/d | n/d | 0 |
| Specimen 15 | pT2c | 5.55 | 54 | 3+4=7 |
| Specimen 16 | pT2c | 6.6 | 51 | 3+4=7 |
| Specimen 17 | pT3a | 6.4 | 43 | 3+3=6 |
| Specimen 18 | pT2c | 7.45 | 54 | 3+4=7 |
| Specimen 19 | pT2c | 26 | 64 | 4+3=7 |
| Specimen 20 | pT2c | 6 | 53 | 3+4=7 |
| Specimen 21 | BPH | 5.5 | 56 | 0 |
| Specimen 22 | pT2c | 6.3 | 55 | 3+4=7 |
| Specimen 23 | pT2c | 7.26 | 54 | 3+4=7 |
| Specimen 24 | pT2c | 7.2 | 62 | 3+4=7 |
| Specimen 25 | pT3bN1 | 42.2 | 47 | 4+4=8 |
| Specimen 26 | BPH | n/d | 42 | 0 |
| Specimen 27 | pT2c | 44 | 64 | 3+4=7 |
| Specimen 28 | BPH | 4.11 | 69 | 0 |
| Specimen 29 | BPH | 10 | 54 | 0 |
| Specimen 30 | Т3АN1 | 15 | 67 | 4+4=8 |
| Specimen 31 | BPH | n/d | n/d | 0 |
| Specimen 32 | pT3b | 18 | 56 | 3+4=7 |
| Specimen 33 | pT2C | 5.4 | 55 | 3+4=7 |
| Specimen 34 | pT2a | 7.9 | 48 | 3+3=6 |
| Specimen 35 | pT3b | 42 | 57 | 4+5=9 |
| Specimen 36 | pT2a | 7.8 | 62 | 3+3=6 |
| Specimen 37 | pT2c | 9.12 | 53 | 3+4=7 |
| Specimen 38 | BPH | 3.42 | 45 | 0 |
| Specimen 39 | pT2b | 19.7 | 72 | 3+4=7 |
| Specimen 40 | pT2a | 4.11 | 54 | 3+3=6 |
| Specimen 41 | pT2b | 7.3 | 59 | 3+4=7 |
| Specimen 42 | pT3a | 8.5 | 48 | 4+4=8 |
| Specimen 43 | BPH | 2.8 | 52 | 0 |
| Specimen 44 | pT2c | 14 | 61 | 3+4=7 |
| Specimen 45 | pT3b | 17.6 | 74 | 5+4=9 |
| Specimen 46 | pT3a | 9.2 | 69 | 4+3=7 |
| Specimen 47 | T2aN0M0 | 27 | 55 | 3+3=6 |
| Specimen 48 | pT2c | 4.9 | 67 | 4+3=7 |
| Specimen 49 | BPH | 2.5 | 60 | 0 |
| Specimen 50 | pT3b | 30 | 49 | 4+4=8 |
| Specimen 51 | pT2b | 11.5 | 71 | 3+3=6 |
| Specimen 52 | pT3a | 18 | 58 | 3+4=7 |
