# Supplementary material for: Myosin 1C isoform A is a novel candidate diagnostic marker for prostate cancer
Source: PLoS One. 2021 May 21;16(5):e0251961. doi: 10.1371/journal.pone.0251961 (PMC8139512; doi:10.1371/journal.pone.0251961)
Supplement: S2 Table — (DOCX) [file pone.0251961.s006.docx]

**S2 Table. Primer sequences for RTqPCR mRNA evaluation**

| Gene symbol | Forward and reverse primer sequence | Amplicon size | Localization |
| --- | --- | --- | --- |
| *YWHAZ* | ACTTTTGGTACATTGTGGCTTCAA  CCGCCAGGACAAACCAGTAT | 94 | 2p25 |
| *HPRT1* | TGACACTGGCAAAACAATGCA  GGTCCTTTTCACCAGCAAGCT | 94 | Xq26 |
| *UBC* | ATTTGGGTCGCAGTTCTTG  TGCCTTGACATTCTCGATGGT | 133 | 12q24 |
| *GAPDH* | TGCACCACCAACTGCTTAGC  GGCATGGACTGTGGTCATGAG | 87 | 12p13 |
| *ACTB* | CTGGAACGGTGAAGGTGACA  AAGGGACTTCCTGTAACAATGCA | 140 | 7p15-p12 |
| *RPL13A* | CCTGGAGGAGAAGAGGAAAGAGA  TTGAGGACCTCTGTGTATTTGTCAA | 126 | 19q13 |
| *B2M* | TGCTGTCTCCATGTTTGATGTATCT  TCTCTGCTCCCCACCTCTAAGT | 86 | 15q21-q22 |
| *TBP* | GAGAGTTCTGGGATTGTACCG  ATCCTCATGATTACCGCAGC | 143 | 6q27 |
| *ALAS1* | AGTGTGAAAACCGATGGAGG  CGATCATACTGAAAAGTGGAAACAG | 140 | 3p21 |
| *SDHA* | TGGTTGTCTTTGGTCGGG  GCGTTTGGTTTAATTGGAGGG | 85 | 5p15 |
| *MYOIC (isoform A)* | GGAGAGATCATCCGTGTGGT  GGACCGATGTAGGTATAAAGAGG | 215 | 17p13 |
