# Supplementary material for: Myosin 1C isoform A is a novel candidate diagnostic marker for prostate cancer
Source: PLoS One. 2021 May 21;16(5):e0251961. doi: 10.1371/journal.pone.0251961 (PMC8139512; doi:10.1371/journal.pone.0251961)
Supplement: S3 Table — (DOCX) [file pone.0251961.s007.docx]

**S3 Table. List of monoclonal antibodies used for the surface staining of prostate cancer and benign hyperplasia specimens.**

| Tube | Marker | Fluorochrome | Clone | Batch # | Manufacturer |
| --- | --- | --- | --- | --- | --- |
| 1 | CD44 | BV421 | BJ18 | B2950966 | BioLegend |
|  | CD90 | BV510 | 5 E10 | B245727 | BioLegend |
|  | CD57 | PerCP-Cy5.5 | HNK-1 | B13219164 | BioLegend |
|  | CD133 | PE | AC133 | 5180313482 | Miltenyi Biotech |
|  | CD24 | APC-H7 | ML5 | 5119894 | BD |
| 2 | CD44 | BV421 | BJ18 | B2950966 | BioLegend |
|  | CD90 | BV510 | 5 E10 | B245727 | BioLegend |
|  | CD10 | FITC | eBioCB-CALLA | E021826 | eBioscience |
|  | CD57 | PerCP-Cy5.5 | HNK-1 | B13219164 | BioLegend |
|  | CD146 | PE | P1H12 | 47560 | BD |
|  | CD38 | PE-Cy7 | HIT2 | B127010 | BioLegend |
|  | CD24 | APC-H7 | ML5 | 5119894 | BD |
| 3 | CD16 | FITC | DJ130c | DJ130c | Dako |
|  | CD54 | PE | HA58 | 35633 | BD |
|  | CD29 | APC | MAR4 | 77396 | BD |
|  | CD38 | PE-Cy7 | HIT2 | B127010 | BioLegend |
| 4 | CD13 | FITC | WM-47 | 0030715 | Dako |
|  | CD166 | PE | 3A6 | 41547 | BD |
|  | CD10 | APC-H7 | HI10a | 4016530 | BD |
