# Supplementary material for: Myosin 1C isoform A is a novel candidate diagnostic marker for prostate cancer
Source: PLoS One. 2021 May 21;16(5):e0251961. doi: 10.1371/journal.pone.0251961 (PMC8139512; doi:10.1371/journal.pone.0251961)
Supplement: S4 Table — (DOCX) [file pone.0251961.s008.docx]

**S4 Table. Surface antigen expression on prostate cells from ex vivo clinical specimens and in vitro model cell lines of malignant and non-malignant origin.** Non-malignant cell line RWPE-1, prostate cancer cell lines PC3, DU145, 22Rv1, LNCaP (top rows) represent in vitro controls. Samples ##2-32 represent prostate carcinomas. Samples ##12-28 represent benign prostate hyperplasia. Data are presented as percent of positive cells (MFI ratio of stained and unstained populations). Positive population gates were set according to unstained controls

| sample/ marker | CD44 | CD24 | CD133 | CD57 | CD90 | CD29 | CD54 | CD146 | CD166 | CD38 | CD10 | CD13 |
| --- | --- | --- | --- | --- | --- | --- | --- | --- | --- | --- | --- | --- |
| RWPE-1 | 99.0% (17.7) | 77.5% (18.3) | 0.3% (1.2) | 0% (1.0) | 0.1% (1.1) | 95.0% (53.2) | 99.0% (46.9) | 84.0% (6.9) | 94.0% (7.7) | 78.0% (15.2) | 0.5% (1.4) | 65.2% (4.7) |
| PC3 | 99.0% (265.5) | 49.0% (8.6) | 0% (1.0) | 0% (1.0) | 0% (1.0) | 97.2% (78.0) | 99.0% (615.6) | 59.1% (3.2) | 99.0% (6.1) | 99.0% (34.7) | 0% (1.0) | 1.2% (1.3) |
| DU145 | 99.0% (81.9) | 52.5% (10.8) | 0.1% (1.1) | 0% (1.0) | 0% (1.0) | 98.2% (33.7) | 99.1% (768.9) | 44.2% (3.9) | 92.0% (6.6) | 98.0% (22.7) | 0% (1.0) | 1.7% (1.6) |
| 22Rv1 | 98.1% (15.3) | 50.3% (3.9) | 0% (1.0) | 0% (1.0) | 0% (1.0) | 96.0% (5.6) | 79.0% (6.1) | 36.3% (2.1) | 99.0% (9.9) | 66.1% (5.2) | 0% (1.0) | 0% (1.0) |
| LNCaP | 0% (1.0) | 57.2% (5.2) | 0% (1.0) | 0% (1.0) | 0% (1.0) | 99.0% (12.1) | 97.1% (6.3) | 19.2% (1.2) | 99.0% (6.1) | 73.4% (21.8) | 88.1% (162.4) | 24.5% (2.2) |
| 2 | 0.3% (17.6) | 0.2% (2.3) | 0.5% (7.4) | 3.5% (20.1) | 25.8% (12.0) |  |  |  |  |  |  |  |
| 3 | 29.0% (6.4) | 23.0% (2.0) | 3.0% (3.8) | 0.9% (8.6) | 29.5% (9.9) |  |  |  |  |  |  |  |
| 4 | 2.4% (20.3) | 40.6% (5.3) | 0.5% (3.2) | 2.0% (6.8) | 1.0% (6.1) |  |  |  |  |  |  |  |
| 5 | 0.6% (19.6) | 45.9% (3.8) | 0% (1.0) | 1.1% (11.5) | 0.9% (6.0) |  |  |  |  |  |  |  |
| 6 | 0.7% (4.6) | 2.0% (3.2) | 0% (1.0) | 0.3% (19.5) | 0% (1.0) |  |  |  |  |  |  |  |
| 7 | 7.7% (13.2) | 16.9% (9.2) | 0.1% (6.2) | 0.4% (10.2) | 0.8% (17.8) |  |  |  |  |  |  |  |
| 8 | 1.8% (15.5) | 43.7% (9.4) | 0.1% (5.7) | 0% (1.0) | 0% (1.0) |  |  |  |  |  |  |  |
| 9 | 2.1% (15.1) | 50.6% (9.7) | 0% (1.0) | 0% (1.0) | 1.6% (19.7) |  |  |  |  |  |  |  |
| 10 | 4.1% (20.3) | 41.0% (1.6) | 0.5% (12.4) | 0% (1.0) | 1.1% (11.0) |  |  |  |  |  |  |  |
| 11 | 0.2% (8.3) | 8.5% (13.7) | 0% (1.0) | 0.2% (11.0) | 0% (1.0) |  |  |  |  |  |  |  |
| 13 | 2.1% (8.6) | 18.5% (4.8) | 0.2% (1.3) | 0% (1.0) | 0.5% (15.0) |  |  |  |  |  |  |  |
| 16 | 3.7% (8.6) | 29.2% (9.4) | 0.7% (1.9) | 0% (1.0) | 0% (1.0) |  |  |  |  |  |  |  |
| 15 | 2.7% (16.3) | 26.0% (3.2) | 0.3% (7.1) | 0% (1.0) | 0% (1.0) | 18.0% (6.2) | 5.3% (14.5) | 7.9% (6.8) | 0.5% (5.2) |  |  |  |
| 17 | 1.0% (10.9) | 50.4% (7.0) | 0.8% (1.3) | 0.1% (8.1) | 0% (1.0) | 36.2% (15.5) | 5.7% (2.0) | 4.6% (1.7) | 2.3% (2.0) | 28.9% (9.8) | 0% (1.0) | 0% (1.0) |
| 18 | 0.8% (13.9) | 64.6% (3.0) | 0.1% (11.0) | 0.1% (12.7) | 0% (1.0) | 0.1% (18.9) | 5.7% (5.5) | 7.8% (7.2) | 3.3% (3.1) | 28.3% (19.7) | 0.7% (8.1) | 0.2% (4.4) |
| 19 | 0.5% (6.0) | 3.8% (4.1) | 0.1% (6.8) | 0% (1.0) | 0% (1.0) | 0% (1.0) | 0.5% (5.3) | 0.4% (3.3) | 0% (1.0) | 2.1% (11.0) | 0.1% (9.1) | 0% (1.0) |
| 22 | 0.2% (9.3) | 4.9% (2.6) | 4.2% (2.5) | 0% (1.0) | 0% (1.0) | 0% (1.0) | 0.7% (6.2) | 2.2% (15.6) | 0.2% (15.6) | 0.3% (17.0) | 0.1% (9.0) | 0.2% (7.3) |
| 23 | 0.4% (7.3) | 10.2% (2.9) | 0% (1.0) | 0% (1.0) | 0% (1.0) | 2.3% (5.9) | 1.1% (8.7) | 1% (7.0) | 0.1% (3.7) | 5.6% (11.3) | 0.1% (13.1) | 0.2% (4.4) |
| 24 | 0.8% (11.3) | 23.6% (2.5) | 0% (1.0) | 0.1% (19.8) | 0% (1.0) | 7.6% (11.7) | 8.6% (7.1) | 5.3% (5.5) | 0.5% (4.8) | 29.9% (14.2) | 0.1% (8.5) | 0% (1.0) |
| 25 | 1% (11.6) | 7.5% (7.6) | 0% (1.0) | 0% (1.0) | 0% (1.0) | 2.1% (9.9) | 0.5% (12.5) | 0.3% (7.2) | 0% (1.0) | 6.1% (20.4) | 0.9% (10.3) | 0% (1.0) |
| 27 | 2.1% (12.2) | 48.7% (2.6) | 0% (1.0) | 0% (1.0) | 0% (1.0) | 7.9% (7.1) | 4.9% (18.9) | 4.6% (11.3) | 2.2% (9.0) | 20.8% (9.3) | 0% (1.0) | 0.1% (5.9) |
| 30 | 4.4% (12.7) | 22.7% (11.6) | 0% (1.0) | 0% (1.0) | 0% (1.0) | 1.5% (9.9) | 14.3% (5.6) | 3.2% (4.9) | 0% (1.0) | 28.6% (7.5) | 1.4%(5.2) | 0% (1.0) |
| 32 | 0.4% (5.5) | 78.8% (3.9) | 2.5% (9.5) | 0.1% (12.2) | 0% (1.0) | 1.1% (12.3) | 1.1% (2.8) | 9.8% (2.5) | 0.1% (4.7) | 11.7% (10.4) | 0.2% (4.7) | 0.2% (4.6) |
| 12 | 2.7% (19.6) | 46.4% (7.9) | 0.4% (3.0) | 0% (1.0) | 0.5% (15.2) |  |  |  |  |  |  |  |
| 14 | 2.3% (16.7) | 41.7% (8.3) | 0.6% (9.4) | 0% (1.0) | 0% (1.0) | 20.4% (5.1) | 8.7% (19.0) | 9.4% (10.2) | 1.8% (9.6) |  |  |  |
| 21 | 1.5% (9.5) | 70.6% (9.3) | 0.1% (5.9) | 0% (1.0) | 0% (1.0) | 18.4% (5.9) | 3.2% (7.6) | 7.8% (4.7) | 0.8% (4.8) | 28.6% (16.0) | 1.2% (4.5) | 0.6% (8.2) |
| 26 | 1.0% (5.3) | 49.9% (5.6) | 1.0% (7.1) | 0% (1.0) | 0% (1.0) | 1.6% (4.2) | 3.7% (6.5) | 1.6% (3.2) | 0.1% (5.6) | 30.4% (19.9) | 0.5% (11.0) | 0.8% (9.9) |
| 29 | 4.8% (7.1) | 50.0% (3.8) | 0.4% (9.1) | 0% (1.0) | 0% (1.0) | 4.0% (4.9) | 12.3% (4.4) | 0.6% (4.2) | 0.2% (4.3) | 43.5% (26.5) | 0.4% (8.9) | 0.2% (10.2) |
| 31 | 1.2% (8.8) | 31.1% (3.4) | 0% (1.0) | 0% (1.0) | 0% (1.0) | 0.1% (9.0) | 6.8% (6.5) | 4.4% (5.8) | 0.2% (5.4) | 23.2% (17.8) | 0.9% (10.7) | 0% (1.0) |
| 28 | 1.0% (32.3) | 62.5% (2.3) | 0.1% (7.2) | 0.1% (13.5) | 0% (1.0) | 13.4% (8.2) | 2.6% (6.9) | 10.2% (5.4) | 0.1% (9.5) | 34.2% (19.5) | 0.4% (7.2) | 0.5% (7.3) |
