# Supplementary material for: Myosin 1C isoform A is a novel candidate diagnostic marker for prostate cancer
Source: PLoS One. 2021 May 21;16(5):e0251961. doi: 10.1371/journal.pone.0251961 (PMC8139512; doi:10.1371/journal.pone.0251961)
Supplement: Attachment — Submitted filename: Response to Reviewers.docx [file pone.0251961.s010.docx]

Dear Dr. Muhammad Saleem,

Thank you for conducting an expert peer review of our manuscript and for your favorable decision concerning our first version. We are submitting a thoroughly revised version for your consideration. We are grateful to both reviewers for their recommendations. We have carefully reviewed the comments and revised the manuscript accordingly. Our complete response and list of the changes follow. Changes to the manuscript are shown in red.

We hope that the revised version is now suitable for publication and look forward to hearing from you soon.

Kind regards,

Aleena Saidova, PhD

Senior Research fellow

School of Biology, Moscow State University

Reviewer 1 Comment 1

It is not very clear in the paper, how the expression of myosin 1C isoform A was evaluated as myosin 1C isoform A has several isoforms. Do authors use any specific antibody? Please include the sequence of primers used in the study.
The gene for Myosin IC encodes three isoforms, termed isoform A, B, and C. Isoforms A contains a unique N-terminal peptide sequence of 35 amino acids that is encoded by an isoform specific exon. This unique exon sequence as well as the unique amino acid sequence allowed us to detect specifically myosin IC isoform A.

The details of the myosin IC isoform A-specific primer that target the isoform A specific exon are included in Table S2.

On protein level, myosin IC isoform A was identified using a monoclonal, isoform A-specific antibody that was raised specifically against the N-terminal isoform A-specific peptide. The antibody is described in Ihnatovych et al., 2012. The antibody has also been licensed to EMD Millipore as an isoform A specific antibody under the name: Anti-MYO1C Antibody, clone B7H8. A better description of this antibody has been added to the “method” section

Reviewer 1 Comment 2

P value were not included in figure 1A
 We have revised figure 1A and included p-value.

Reviewer 1 Comment 3

Since authors believe that myosin 1C isoform A could be used as a Biomarker. Does author’s tried to find the expression of myosin 1C isoform A in patent serum or urine.

The possibility that the reviewer raises is an important one for further development of this biomarker. While detection of myosin IC isoform A in serum or urine is outside the scope of this paper, which for the first time clearly establishes clinical relevance of this marker and focuses on its use in analyzing biopsy material, we are planning to assess its use in non-invasive diagnostics in the near future and have included the following text in the Discussion:

“Validation of biomarkers suitable for non-invasive diagnostics using patients’ serum and urine is an important direction in the current research on PCa (see, e.g., <https://pubmed.ncbi.nlm.nih.gov/23308129/>, <https://pubmed.ncbi.nlm.nih.gov/30129068>, <https://pubmed.ncbi.nlm.nih.gov/33256740/>). In the light of the present findings that establish utility of myosin IC isoform A in analyzing biopsy samples and our recent results (<https://pubmed.ncbi.nlm.nih.gov/28814772/>) that demonstrated presence of myosin IC isoform A in exosomes secreted by PCa cells, assessing diagnostic applications of this isoform’s detection in serum or urine is a logical next step in validation of this biomarker for clinical use.”

Reviewer 2 Comment 1

Add vendors' names, city, and country of origin for each instrument and software.

We have revised the Materials and Methods and added vendor details for instruments and software

Reviewer 2 Comment 2

Authors need to increase the clinical sample number for a conclusive result.

We have increased the clinical dataset on 15 samples, that allowed us to discriminate II and III clinical stages based on isoform A expression.

Reviewer 2 Comment 3

I am wondering why the authors analyzed only cancer stage II and III samples. They should include stage I and Stage IV.

Stage I is almost impossible to include since biopsies are very rarely done at this stage. Similarly, about stage IV – usually the patients are coming to the hospital at earlier stage (II or III) and biopsy cannot be postponed to follow cancer progression due to ethical reasons. Moreover, the common protocol at stage IV is chemotherapy to reduce the cancer mass that would be then classified as stage III.

Reviewer 2 Comment 4

Authors must explain how their Myosin 1C isoform A is a better diagnostic marker than previously published biomarkers (PMID: 23308129; PMID: 33256740; PMID: 33113419; PMID: 33058520)

We have added the following passage to the Discussion, which references the literature indicated by the reviewer:

“Validation of biomarkers suitable for non-invasive diagnostics using patients’ serum and urine is an important direction in the current research on PCa (see, e.g., <https://pubmed.ncbi.nlm.nih.gov/23308129/>, <https://pubmed.ncbi.nlm.nih.gov/30129068>, <https://pubmed.ncbi.nlm.nih.gov/33256740/>). In the light of the present findings that establish utility of myosin IC isoform A in analyzing biopsy samples and our recent results (<https://pubmed.ncbi.nlm.nih.gov/28814772/>) that demonstrated presence of myosin IC isoform A in exosomes secreted by PCa cells, assessing diagnostic applications of this isoform’s detection in serum or urine is a logical next step in validation of this biomarker for clinical use. Expression of myosin IC isoform A, according to the data presented here, is a robust and differentiating feature of clinical samples from the different stages of the disease, and thus compares favorably with markers that are detected in small subgroups of patients (e.g., the recently characterized isoform of NCOR1, see PMID: 33058520). Assembly of biomarker panels with high collective diagnostic capacity is another direction of current work in the field (see, e.g., <https://pubmed.ncbi.nlm.nih.gov/33256740/>). The ability to differentiate stages of PCa progression that is demonstrated here raises the likelihood of eventually including myosin IC isoform A in a panel of biomarkers that will be capable of accurately and predictively diagnosing PCa and the likely course of the disease.”
